# Genetic Parameters of Birth Weight and Weaning Weight and Their Relationship with Gestation Length and Age at First Calving in Hanwoo (*Bos taurus coreanae*)

**DOI:** 10.3390/ani10061083

**Published:** 2020-06-23

**Authors:** Bryan Irvine Lopez, Kier Gumangan Santiago, Kangseok Seo, Taejoon Jeong, Jong-Eun Park, Han-Ha Chai, Woncheoul Park, Dajeong Lim

**Affiliations:** 1 Division of Animal Genomics and Bioinformatics, National Institute of Animal Science, Rural Development Administration, Wanju 55365, Korea; irvinelopez@korea.kr (B.I.L.); jungtaejoon@naver.com (T.J.); jepark0105@korea.kr (J.-E.P.); hanha@korea.kr (H.-H.C.); wcpark1982@korea.kr (W.P.); 2 Department of Animal Science and Technology, Sunchon National University, Suncheon 57922, Korea; santiagokier2015@gmail.com (K.G.S.); sks@sunchon.ac.kr (K.S.); 3 Department of Animal Science, College of Agriculture, Central Luzon State University, Science City of Muńoz 3120, Philippines

**Keywords:** birth weight, weaning weight, reproductive traits, genetic parameters, heritability, Hanwoo cattle

## Abstract

**Simple Summary:**

Hanwoo cattle is considered one of the most economically important species and sources of nutrition for Koreans. Thus, both the reproductive performance and growth traits play an important role in the continuous development and productivity of Hanwoo farming. Therefore, to improve beef production and the profitability of Hanwoo farming, estimations of genetic parameters for birth weight (BW) and weaning weight (WW) and their relationship with age at first calving (AFC) and gestation length (GL) are necessary to understand and improve their responses to selection. Thus, this study estimated the variance components, heritability estimates of birth weight (BW) and weaning weight (WW) and their genetic and phenotypic relationship to age at first calving (AFC), and gestation length (GL). Results revealed a moderate and high heritability estimate for BW and WW, which suggests a sluggish and rapid response of these traits to selection, respectively. The moderate and high genetic correlation between BW and reproductive traits (AFC and GL) revealed that the selection of a heavier BW might result in a longer AFC and GL. Although the genetic correlation for BW and AFC was moderate and positive, the phenotypic correlation of close to zero may indicate that the phenotypic expression for these traits is likely to be influenced by the genotype-environment interaction (GxE). Moreover, the genetic and phenotypic correlation between WW and reproductive traits (AFC and GL) indicates that the selection of a heavier WW may not influence the AFC and GL of Hanwoo cow. These estimated genetic parameters and correlations for the studied traits can be utilized for genetic breeding programs of Hanwoo cattle.

**Abstract:**

Hanwoo is one of the most economically important animal species in Korea due to its significant contribution to nutrition. However, the current selection index only focuses to improve carcass traits of Hanwoo. Thus, this study aimed to estimate the genetic parameters of birth weight (BW) and weaning weight (WW) and their genetic and phenotypic relationship to the age at first calving (AFC) and gestation length (GL) of Hanwoo. The genetic parameters for birth weight (BW) and weaning weight (WW) were estimated using the data obtained from 52,173 and 35,800 Hanwoo calves born from February 1998 to March 2017, respectively. Further, these data were used to determine their genetic and phenotypic correlation to age at first calving (AFC) and gestation length (GL). The heritability estimates of BW and WW and correlation coefficients were obtained using the average information restricted maximum likelihood (AIREML) procedure, fit in single and two-trait linear animal models. The estimated direct heritability for BW and WW was moderate (0.22 ± 0.02) and high (0.51 ± 0.03), respectively, while the maternal heritability for both traits was 0.12 ± 0.01 and 0.17 ± 0.01, respectively. The genetic correlation of BW and reproductive traits (AFC and GL) showed a moderate and high positive correlation coefficient of 0.33 ± 0.06 and 0.53 ± 0.02, respectively, while close to zero and low positive phenotypic correlations of 0.06 ± 0.01 and 0.21 ± 0.06 were also observed between the correlated traits, respectively. For the correlation analysis between WW and AFC, both the genetic and phenotypic correlation showed close to zero values of 0.04 ± 0.06 and −0.01 ± 0.01, respectively. Meanwhile, the genetic and phenotypic correlation between WW and GL showed low and negative correlations of −0.09 ± 0.06 and −0.09 ± 0.01, respectively. These obtained estimated variances for BW and WW and their corresponding genetic and phenotypic correlation to AFC and GL can be used as information for genetic improvement and subsequent economic improvement of Hanwoo farming.

## 1. Introduction

Hanwoo (Korean cattle) was previously used for farming, transportation, and religious sacrifice [1]. However, with the rapid progress of the Korean economy, their original purpose gradually changed into a full-scale meat-type cattle, and it is now one of the most economically important animal species in Korea due to its significant contribution to the nutrition of Koreans [1]. In the past decade (2005–2015), an overall increase of 44% in the Hanwoo population was observed [2]. Further, improvement in the Hanwoo performance was observed such as an increase in yearling weight (YW) and carcass weight (CW) for the past 15 years (1996–2000), and improvement of the eye muscle area (EMA) and marbling score (MS) for the past 10 years (2001 to 2010) [3]. These observations indicate successful and efficient breeding and feedings system approach of the Korean government. However, the overall increase in the Hanwoo population was attributed only to large-scale farms due to the observed reduction in small-scale Hanwoo farmers as a result of financial loss or conversion into larger farms [4]. Since small-scale farmers are mostly calf producers (cow-calf production), their reduction leads to a potential decline in calves supply which might cause shortages to both medium and large farms venturing into feedlots and composite (feedlots with cow-calf) systems [4]. Though the evident improvement to the Hanwoo industry indicated efficiency, the Korean government remains keen to conduct researches on reproductive, carcass, and meat quality traits that benefit both Hanwoo farmers and consumers.

The birth weight (BW) plays a very good economic indicator in the beef cattle industry and is usually the first trait being measured in a calf [5]. This variable was considered by many researchers as a good economic indicator as it was positively associated with post-weaning daily weight gain [6] and matured weight [7]. Contrastingly, previous studies concluded that BW is a valuable predictor of dystocia (difficulty in giving birth) in cow, perinatal mortality in calves [8,9], and a prolonged calving interval [10]. Aside from BW, the selection of a heavier weaning weight (WW) was also reported to prolong the gestation length (GL) [10] and provide a favorable response in age at first calving (AFC) [11]. In a study conducted by Santana et al. [12], it was reported that growth traits and reproductive traits can be improved simultaneously in Nellore cattle. At present, studies about the genetic and phenotypic correlations between BW and WW to the reproductive traits of cattle are still scarce. Therefore, research must be conducted to examine the relationship between BW and WW to the reproductive traits of cattle such as AFC and GL. However, due to the numerous differences in cattle breeds, it is better to conduct breed-specific research that may provide potential information about the most appropriate BW and WW that could clarify earlier claims. Thus, this study aimed to estimate the genetic parameters of BW and WW and their genetic and phenotypic relationship to the AFC and GL of Hanwoo.

## 2. Materials and Methods

### 2.1. Animals and Trait

The data on birth weight (BW) and weaning weight (WW) were collected from 52,173 and 35,800 Hanwoo calves born from the period of February 1998 to March 2017, respectively. The BWs of calves were weighed and recorded within 24 h post-partum. Subsequently, these calves were raised from their dam until 90 days of age to determine the WW of each calf. The Hanwoo calves in this study were produced through the purebred mating system done using artificial insemination of semen collected from bulls that were initially selected based on their performance and progeny carcass traits. There were also two reproductive traits included in this study, particularly age at first calving (AFC) and gestation length (GL), with total records of 14,258 and 51,303, respectively. The AFC was determined by determining the birthdate of the heifer and the date of its first calving, while the GL was computed using the last date of insemination and the calving date. Additional details for AFC and GL can be found in our previous studies [13]. Furthermore, the 9 generation-deep pedigree data consisted of 66,736 animals with 543 bulls, and 20,006 dams were included in the analysis. All of these data were gathered from different herds in nine provinces across South Korea and were acquired through an existing database. The approval from the Animal Care and Use Committee was not needed in this study since all data were acquired from the database. The data retained after the quality check includes BW with a value ranging from 17 to 38.30 kg, WW between 31 and 182 kg, AFC between 555 and 961 days, and GL between 271 and 299 days. The sample population, means, standard deviation, minimum, and maximum values for each trait are presented in Table 1.

### 2.2. Statistical Analysis

Before the genetic parameter estimation, the non-genetic factors were initially analyzed to determine the significant (*p* < 0.05) fixed effects and interactions to be included in the model. Among the non-genetic effects, the following fixed effects were tested for each trait: herd where the animal was raised, birth year, birth month, birth season, parity of the cow, sex of the calf, and age of dam at calving. The analysis was done by way of ordinary least squares using the MIXED procedure of the SAS software (SAS Institute, Inc., Cary, NC, USA, 2012). Results of the initial analyses revealed that the herd (98 levels), sex (2 levels), birth year and month (228 levels), and parity of the cow (5 levels) were significant and can be used as fixed effects for BW and WW. In the correlation analysis of BW and WW to reproductive traits, both AFC and GL were analyzed as traits of the calf, with herd and birth year-month of the dam as fixed effects for the earlier trait, while herd, calf birth year-month, and parity were analyzed as fixed effects for the latter trait.

Genetic parameters and standard errors were estimated using the average information restricted maximum likelihood (AIREML) method. Analyses were conducted using the AIREMLF90 software [14] considering the convergence criterion of 10^−15^. A single-trait model analysis was fitted to determine the variance components of BW and WW, while a two-trait analysis using a similar model was fitted to determine the genetic correlation of BW and WW to AFC and GL. The general animal model was

$$y=Xb+Z_{1}a+Z_{2}m+e$$

where **y** is the vector of observations for each trait; **b** is the vector of all fixed effects; **a** is the vector of random direct additive genetic effects of the animals; **m** is the vector of random maternal genetic effects; and **e** is the vector of random residual errors associated with the observations. Meanwhile, **X**, **Z_1_**, and ***Z*_2_** are incidence matrices related to fixed, additive genetic, and maternal genetic effects, respectively. It was assumed that E[y]=Xb; the direct additive genetic, genetic maternal, and residual effects were normally distributed with means of zero and $\mathrm{Var}\left(a\right)=A\;⊗\;S_{a}$; $\mathrm{Var}\left(m\right)=A\;⊗\;S_{m}$; $\mathrm{Var}\left(e\right)=I\;⊗\;S_{e}$; in which **S_a_** is the direct additive genetic covariance matrix; **S_m_** is the maternal genetic variance matrix; **S_e_** is the residual covariance matrix; **I** is the identity matrix. The covariance between the direct and maternal genetic effects was set to zero. Moreover, similar in our previous study [13], the additive genetic and residual effects were used as random effects for the analysis of AFC while additive genetic, permanent environmental, and residual effects were used for GL. 

## 3. Results

### 3.1. Heritability Estimates for Birth Weight and Weaning Weight

The direct and maternal heritability estimates and variance components for birth weight (BW) and weaning weight (WW) using a single-trait analysis are presented in Table 2. In terms of variances, the maternal variances of 1.12 ± 0.06 and 65.76 ± 6.66 for BW and WW, respectively, were consistently lower compared with their corresponding additive genetic variance of 2.09 ± 0.15 and 200.31 ± 20.16. The direct heritability for BW of 0.22 ± 0.02 was moderate, while for WW, a high heritability estimate of 0.51 ± 0.01 was observed. In terms of maternal heritability estimates, the obtained estimate for BW of 0.12 ± 0.01 was slightly lower compared with the maternal heritability estimate of 0.17 ± 0.01 for WW.

### 3.2. Genetic and Phenotypic Correlations

The genetic and phenotypic correlations between BW and WW to reproductive traits (AFC and GL) are shown in Table 3. Results of the correlations analysis revealed a moderate positive genetic correlation of 0.33 ± 0.06 and low phenotypic correlation of 0.06 ± 0.01 between BW and AFC, while for WW and AFC, a close to zero genetic correlation and phenotypic correlation of 0.04 ± 0.07 and −0.01 ± 0.01 were observed, respectively. Meanwhile, a highly positive genetic correlation of 0.53 ± 0.02 and a moderate positive phenotypic correlation of 0.21 ± 0.06 was observed between BW and GL, while low and negative genetic and phenotypic correlation coefficients of −0.09 ± 0.06 and −0.09 ± 0.01 were observed between WW and GL, respectively.

## 4. Discussion

The results obtained in this study were compared to numerous literature with almost similar model structures except only for studies with non-zero assumptions of the covariance value between the direct additive and maternal variance, and the inclusion of a permanent environmental effect, which was not included in our model since only a few cows have twin calves.

### 4.1. Heritability Estimates for Birth Weight

Birth weight was considered a very good economic indicator in the beef cattle industry [5] as it was positively associated with post-weaning gain [6] and mature weight [7]. Contrariwise, calf birth weight was also identified to be a crucial factor for calving ease and directly associated with dystocia and perinatal mortality [8,9]. Therefore, determining the heritability estimates of birth weight in a cattle herd might boost economic productivity by improving growth traits and limiting the associated birth weight problems.

The observed direct and maternal heritability estimates for BW of 0.22 ± 0.02 (Table 3) and 0.12 ± 0.01, respectively, were comparable to 0.20 and 0.09 obtained by Choi et al. [15] in Hanwoo calves using a model similar to this study, except only for a non-zero covariance assumption between the additive genetic and maternal effects. Contrastingly, numerous researchers including Kars et al. [16] (0.41, Nguni cattle stud), Mujibi et al. [17] (0.46, Charolais), Martínez-González et al. [18] (0.59, Nellore), and Tawah et al. [19] (0.39, Gudali; 0.65, Wakwa Cattle) reported higher direct heritability estimates compared with our findings. Meanwhile, the obtained maternal heritability for birth weight in this study was lower compared with those observed by Kars et al. [16] and Martínez-González et al. [18] of 0.16 and 0.17, respectively. In addition, Tawah et al. [19] reported a lower maternal heritability of 0.06 in Gudali cattle and a higher heritability of 0.22 in Wakwa cattle. According to Martínez-González et al. [18], once BW is involved in a breeding program, the additive genetic and maternal effects must be considered in constructing the selection indices.

In this study, the covariance value between the direct and maternal effects was set to zero. This assumption was different with Choi et al. [15], Kars et al. [16], Mujibi et al. [17], Martínez-González et al. [18], and Tawah et al. [19] (Gudali and Wakwa Cattle), who observed covariance values of 0.25, −1.69, −1.31, −0.44, −0.99, and −3.38, respectively. Such differences in the covariance assumptions might bring the lower direct heritability estimate in this study and might bring higher direct heritability for those researches [16,17,18] who obtained a negative covariance value. This observation was supported by Kars et al. [16], who reported that negative covariance between direct and maternal heritability may result in a reduction in the total heritability. Conversely, the positive and close to zero covariance value observed by Choi et al. [15] in Hanwoo cattle resulted in comparable direct and maternal heritability estimates with the findings in this study.

Alongside the covariance assumption, breed differences and large unexplainable residual variances also bring discrepancies to heritability estimates. Specifically, the obtained residual variance in this study contributes to about 66.21% of the total phenotypic variance which was 10.40% to 19.31% higher than those observed by Kars et al. [16], Mujibi et al. [17], Martínez-González et al. [18], and Tawah et al. [19] (Wakwa Cattle), and 1.70% to 3.24% lower than those observed by Tawah et al. [19] (Gudali Cattle) and Choi et al. [15], respectively.

Despite differences, our findings suggest that Hanwoo’s birth weight will sluggishly respond to selection. However, strict caution must be considered when selecting calves with heavy birth weight, since it might increase dystocia cases and subsequent perinatal mortality. Further, our results indicate that maternal effect plays a very important role in cattle BW. Similarly, Chud et al. [20] stated that the genetic potential of the dam also influences the calf’s performance for traits such as BW, WW, and GL.

### 4.2. Heritability Estimates for Weaning Weight

In here, the obtained direct and maternal heritability estimates for weaning weight of 0.51 ± 0.03 and 0.17 ± 0.01 were close to the observation of Gutiérrez et al. [21] in Asturiana de los Valles beef cattle of 0.45 and 0.14, respectively, but different compared with 0.10 (direct) and 0.27 (maternal) observed by Choi et al. [15] in Hanwoo calves, respectively. Although Choi et al. [15] used various models, the closest model structure with our study (except for a zero covariance value) still obtained opposite findings, which might be attributed to the larger residual variance of approximately 80% of the total phenotypic variance compared with only 32% observed in this study.

Meanwhile, numerous researchers who included a permanent maternal environment effect reported lower direct heritability and comparably low maternal heritability estimates. Chud et al. [20] and Boligon et al. [22] obtained lower direct and maternal heritability estimates for Nellore cattle. According to Chud et al. [20], the inclusion of maternal and permanent environmental effects avoids the overestimation of direct additive genetic effects for traits like BW, WW, and GL.

Furthermore, the maternal heritability estimates observed in this study fall within the range of 0.10 to 0.17 observed by Schiermiester et al. [23], Wasike et al. [24] (Boran cattle), and Phocas and Laloë [25] (French specialized beef cattle breeds). This observation indicates that the maternal dependence of a calf is usually observed from birth until weaning, thus resulting in a significant contribution of maternal effects to phenotypic variance [22]. Further, Meyer [26] reported that ignoring maternal effects in growth traits until weaning will result in inflation of direct heritability.

Although differences in estimates were observed, our observation suggests that the differences in the direct heritability estimates can be attributed mainly to breed differences, residual variances, and variations to model structure such as the inclusion of maternal permanent environmental effects. Moreover, our findings indicate that weaning weight cannot be influenced by its genetic potential alone but also with the maternal effects of its dam. Further, the high direct heritability estimates observed for WW suggest a rapid response to selection and might bring an immediate advantage to calves to gain a heavier bodyweight at older ages.

### 4.3. Genetic and Phenotypic Correlations

The obtained genetic correlation (r_g_) between BW and AFC of 0.33 ± 0.064 was lower compared with 0.77 observed by Bekele et al. [27] in Fogera cattle, and opposite compared with −0.17 reported by Bourdon and Brinks [28] in herds consisting of Red Angus, Angus, and Hereford. In terms of the phenotypic correlation (r_p_) between BW and AFC, the obtained value in this study of 0.06 ± 0.01 was slightly lower than 0.12 observed by Bourdon and Brinks [28] but far lower compared with 0.24 reported by Bekele et al. [27]. According to the latter researchers, the positive r_g_ between BW and AFC indicates that selecting a calf with heavy growth traits including BW results in a longer AFC [27], while a negative relationship indicates a favorable relationship between BW and early production [28]. Meanwhile, the low r_p_ between these traits can be attributed to the relationship between the additive genetic effects and non-additive genetic effects (environmental factors). According to Wakchaure et al. [29], the genotype by environment (GxE) interaction can be determined at the phenotypic level, and can also be evaluated using the level of correlation, wherein a high genetic correlation means a slight GxE interaction effect, while a low genetic correlation indicates a strong influence of the GxE interaction to the animal performance. 

Meanwhile, the observed r_g_ between BW and GL of 0.53 ± 0.02 (Table 3) was comparable to 0.49 observed by Kemp et al. [30] in Simmental cattle, which also suggests that selecting animals with short GL results in calves with low a BW and easier calving. Contrariwise, a smaller and positive r_g_ was observed by Chud et al. [20] in Nellore (0.19), Hwang et al. [10] in Hanwoo (0.42–0.45), and a high and negative correlation of −0.84 by Bekele et al. [27] in Fogera cattle. Furthermore, the obtained r_p_ between BW and GL in this study of 0.21 ± 0.06 (Table 3) was similar to the findings of Kemp et al. [30] of 0.21, but lower compared with the observation of Hwang et al. [10], with a value ranging from 0.29 to 0.31. According to Hwang et al. [10], the positive genetic and phenotypic relationship between GL and BW indicates that longer GL might result in a heavier calf at birth, which may lead in a faster growth rate during the suckling period but might also lead to higher cases of dystocia and longer calving intervals.

The low and near to zero r_g_ between WW and AFC of 0.04 ± 0.07 was close to −0.02 observed by Segura-Correa [31] in Brown Swiss and comparably lower than the findings of Pereira et al. [32] of 0.10 in Nellore. The earlier researcher concluded that the close to zero correlation coefficient indicates that increasing the WW will not result to any change in AFC, while the latter concluded that a low to moderate positive r_g_ between WW and reproductive traits (AFC, GL and days to calving) indicates that to avoid low reproductive performance, the practice of increasing adult size should not be recommended. Meanwhile, Boligon et al. [22], Boligon et al. [33], and Talhari et al. [11] reported higher negative r_g_ (between WW and AFC) of −0.20 and −0.16 in Nellore and −0.12 in female Canchim cattle, respectively. According to these authors, the selection to increase WW and other growth traits result in a favorable response and possible reduction in AFC. Moreover, the obtained r_p_ between WW and AFC in this study did not differ from zero (−0.01 ± 0.01) but was found to be lower when compared with those observed by Boligon et al. [33] and Segura-Correa [31] of −0.18 in Nellore and −0.29 in Brown Swiss, respectively.

Meanwhile, the r_g_ and r_p_ observed between WW and GL in this study were found to have the same near to zero value of −0.09 ± 0.06 and −0.09 ± 0.01, respectively. The observed r_g_ in this study was comparable to 0.02 ± 0.06 observed by Chud et al. [20] in Nellore cattle. Contrastingly, Hwang et al. [10] reported a moderate and positive r_g_ ranging from 0.21 to 0.22, and low and positive r_p_ ranging from 0.08 to 01.0 between WW and GL of Hanwoo cattle. The observed similar r_g_ and r_p_ between WW and GL in this study might indicate that both traits were independent of each other. This claim was in agreement with Cervantes et al. [34], who reported that except for BW, the GL is genetically independent of the majority of economically important beef cattle traits.

To further support earlier claims, the residual correlation (r_e_) coefficients between growth traits and reproductive traits considered in this study were also presented in Table 3. The obtained r_e_ coefficients between BW and reproductive traits (AFC and GL) were both low and positive, while negative and close to zero r_e_ coefficients were observed between WW and reproductive traits (AFC and GL). According to Kluska et al. [35], a close to zero and low r_e_ indicates that the traits of interest were independently influenced by environmental and non-additive effects.

Despite the differences observed in the correlation coefficients, our findings indicate that the selection of a higher BW might prolong AFC under the appropriate environmental conditions. Although a minimal increase to AFC is expected, it still needs consideration since a longer AFC than normal represents the not income generation period for heifers [36]. This interpretation was supported by Bourdon and Brinks [28], who concluded that a negative correlation between growth traits and age at first calving is more acceptable for it indicates the favorable relationship between growth traits and early production. Furthermore, the moderate to high positive genetic r_p_ between BW and GL indicates that cows with long GLs tend to have a heavier BW since the fetus has more time to gain weight [28], which might eventually increase cases of dystocia [10]. Further, a longer GL causes a year to year increase in the calving interval [37], which is considered an indicator of unproductiveness in the cattle herd. Meanwhile, the correlation observed between WW and reproductive traits (AFC and GL) in this study indicates that the selection of WW will not influence the AFC and GL of Hanwoo cow.

In general, our findings suggest that the selection of a breed-specific optimum birth weight might limit the possible consequences of having a longer AFC and GL. Also, it is suggested to further investigate the relationship of WW to GL since the low and negative correlation might still shorten or prolong the GL of Hanwoo cow.

## 5. Conclusions

The heritability estimates observed for the BW and WW traits of Hanwoo indicates a slow and rapid response to selection, respectively. Further, evidence revealed that the genetic potential of the dam perceived through maternal heritability can influence calf traits like BW and WW. Thus, ignoring maternal effects for these traits will surely result in the inflation of direct heritability. However, genetic improvement for these traits through selection requires strict caution since it may positively or negatively affect equally important traits. The results of the genetic correlation between BW and reproductive traits (AFC and GL) indicate that selecting a calf with a heavier BW might result in a longer AFC and GL. Although there was a moderate and positive genetic correlation between BW and AFC, the low phenotypic correlation may indicate that GxE interaction might influence thephenotypic expression of these traits. Meanwhile, the genetic and phenotypic correlation between WW and reproductive traits (AFC, and GL) indicates that the selection of a heavier WW may not influence the AFC and GL of Hanwoo cow. These obtained variance components, heritability estimates, and correlation coefficients for growth and reproductive traits in this study can be utilized in future researches which aims to improve the Hanwoo cattle industry.

## Figures and Tables

**Table 1:** Descriptive statistics of the birth weight (BW), weaning weight (WW), age at first calving (AFC), and gestation length (GL) of Hanwoo cattle.

Trait	*n*	Mean	SD	Min	Max
BW	52,173	27.41	3.59	17.00	38.30
WW	35,800	92.63	22.20	31.00	182.00
AFC	14,258	752.35	69.36	555.00	961.00
GL	51,303	286.15	5.29	271.00	299.00

BW, birth weight; WW, weaning weight; AFC, age at first calving; GL, gestation length.

**Table 2:** Variance components, direct and maternal heritability estimates for birth weight and weaning weight of Hanwoo calves.

Trait	$\sigma_{a}^{2}$	$\sigma_{m}^{2}$	$\sigma_{e}^{2}$	$\sigma_{p}^{2}$	$h_{m}^{2}$	$h_{a}^{2}$
Birth Weight	2.09 ± 0.15	1.12 ± 0.06	6.29 ± 0.09	9.50 ± 0.08	0.12 ± 0.01	0.22 ± 0.02
Weaning Weight	200.31 ± 20.16	65.76 ± 6.66	127.20 ± 10.29	393.27 ± 16.02	0.17 ± 0.01	0.51 ± 0.03

$\sigma_{a}^{2}$, additive genetic variance; $\sigma_{m}^{2}$, maternal genetic variance; $\sigma_{e}^{2}$, residual variance; $\sigma_{p}^{2}$, phenotypic variance; $h_{m}^{2}$, maternal heritability; $h_{a}^{2}$, direct heritability.

**Table 3:** Genetic, phenotypic, and residual correlations of birth weight (BW) and weaning weight (WW) to age at first calving (AFC) and gestation length (GL).

Traits	AFC			GL		
	r_g_	r_p_	r_e_	r_g_	r_p_	r_e_
BW	0.33 ± 0.06	0.06 ± 0.01	0.08 ± 0.01	0.53 ± 0.02	0.21 ± 0.06	0.16 ± 0.06
WW	0.04 ± 0.07	−0.01 ± 0.01	−0.06 ± 0.01	−0.09 ± 0.06	−0.09 ± 0.01	−0.09 ± 0.01

r_g_, genetic correlation; r_p_, phenotypic correlation; r_e_, residual correlation.
